# Tracking Demographic Movements and Immunization Status to Improve Children's Access to Immunization (TDM-IAI): Protocol for a Field-Based Randomized Controlled Trial

**DOI:** 10.2196/21734

**Published:** 2021-02-08

**Authors:** Jérôme Ateudjieu, Ndinakie Martin Yakum, André Pascal Goura, Etienne Guenou, Landry Bita’a Beyala, Lapia Amada, Isabelle Ngoche, Frank Forex Kiadjieu, Charlette Nangue, Elvis Briand Soukep Djosseu, Bruno Kenfack

**Affiliations:** 1 Meilleur Accès aux soins de Santé (MA SANTE) Yaoundé Cameroon; 2 Division of Health Operations Research Ministry of Public Health Yaoundé Cameroon; 3 Department of Public Health, Faculty of Medicine and Pharmaceutical Sciences University of Dschang Dschang Cameroon; 4 Department of Microbiology Faculty of Sciences University of Buea Buea Cameroon; 5 Meilleur Accès aux soins de Santé (MA SANTE) Foumban Cameroon; 6 Faculty of Health Sciences University of Buea Buea Cameroon; 7 University Teaching Hospital Yaoundé Cameroon; 8 District Medical Office Foumban Health District West-Cameroon Foumban Cameroon; 9 Department of Gynecology and Obstetrics Faculty of Medicine and Pharmaceutical Sciences University of Dschang Dschang Cameroon; 10 Department of Gynecology and Obstetrics Dschang District Hospital West-Cameroon Dschang Cameroon

**Keywords:** immunization status, coverage: completeness, timeliness, EPI vaccines, children under five, Foumban, Cameroon, vaccines, infectious, immunization

## Abstract

**Background:**

In Cameroon, the coverage, completeness, and timeliness of the Expanded Programme on Immunization (EPI) vaccines administration in children have remained heterogeneous and below the national and districts targets in several districts. In an effort to solve this problem, many interventions have been tested but none has shown significant improvement of the situation.

**Objective:**

This trial aims to test whether involving Community Volunteers to assess children vaccination status and demographic movements and using recorded data to plan catch-up immunization sessions can improve children vaccination timeliness, completeness and coverage.

**Methods:**

Communities of the Foumban Health district, West region of Cameroon will be selected and assigned to either intervention or control groups using a restricted randomization of 2. In the intervention group, one Community Volunteer per community will be trained to visit households and record EPI-targeted children in a register, record their demographic movements, and assess their immunization status monthly for a year. The information recorded will be snapped and sent to the competent health center immunization team through WhatsApp. These will be used to plan and implement monthly community catch up immunization sessions in collaboration with the community volunteer. In the control group, the routine immunization sessions will be conducted with health centers organizing either weekly vaccination sessions for communities situated not farther than 5 kilometers away from the health facility or monthly vaccination sessions in communities situated more than 5 kilometers away from the health center. Baseline, mid-term and end-line surveys will be conducted to assess and compare immunization coverage, timeliness, and completeness.

**Results:**

Funded in 2018, data collection started in 2018 and has been completed. Data analysis and reporting are ongoing.

**Conclusions:**

This trial is expecting to test an innovative approach to improving children’s immunization timeliness, completeness and coverage of immunization by tracking EPI targeted population vaccination status and denominator at household level and building collaboration between the community and health facilities vaccination teams to organize monthly community-based response vaccination sessions. This intervention is expected to improve children sustainable access to EPI vaccination as it offers assessing and responding to their immunization needs at monthly basis using low cost local human resources.

**Trial Registration:**

Pan African Clinical Trials Registry ID PACTR201808527428720; tinyurl.com/u058qnse

**International Registered Report Identifier (IRRID):**

DERR1-10.2196/21734

## Introduction

Vaccines save lives cheaply, and many countries have adopted a number of antigens to be administered to children and pregnant women under the Expanded Programme on Immunization (EPI). The program has been successfully implemented in many contexts but in many others, vaccination performance in terms of coverage, completeness, and timeliness remains low and is associated with outbreaks of vaccine preventable diseases [1-3].

In Cameroon, 11 vaccines are planned to be administered to children and pregnant women through the EPI [4]. These vaccines are routinely offered in health facilities on a scheduled day weekly and monthly in communities with limited access to health care. Community-based sessions are outreach activities organized by health facility staff in collaboration with community volunteers. Due to limited resources (human, financial, vaccine supply and cold chain, transportation, and power supply) on one hand and limited knowledge of caregivers and their demographic movements on the other, many children fail to receive their planned vaccine doses, be vaccinated on time, or complete their vaccination as required by the national EPI program [1,5].

In 2018, the Demographic Health Survey conducted at the household (HH) level reported 86.7%, 71.5%, and 65.3% rates of vaccination for bacille Calmette-Guérin (BCG), Diphtheria-Pertussis-Tetanus and Hepatitis B + Hemophilus Influenzae type b (DPT-Hi+Hb3), and measles, respectively, with a zero-dose proportion of 9.7% [6]. Many other studies and reports highlighted heterogeneous immunization coverages and high dropout rates in the child vaccination cascade [7,8]. The association between missing planned vaccination doses and the incidence of EPI-preventable diseases is yet to be investigated in Cameroon. In other settings, low vaccination coverage, timeliness, and completeness rates have been consistently reported to be associated with a high incidence of EPI vaccine–preventable diseases (EPI-PD) [9]. Most cited factors associated with low immunization coverage and incomplete vaccination status of children include maternal socioeconomic status, forgetting the vaccination schedule, limited access to health care services, population health care–seeking behaviors, perception of vaccination, misestimating the targeted population, migration, and demographic movement [10].

Strategies have been tested in many countries to reduce missed opportunities of vaccination and improve access to vaccines. Those frequently reported to have shown some positive impact include providing information on immunization to parents and community members, offering memory cards specifically designed for immunization, offering vaccines through proximity vaccination sessions with or without incentives, identifying unvaccinated children in home visits and referrals to health facilities, and integration of immunization services with other services [11,12].

From previous experience with EPI activity supervision, we noticed that many children and pregnant women miss vaccinations during the scheduled periods because of short- or long-term travel. In the national immunization guidelines, no procedure has been planned to catch-up and reduce the time gaps between the recommended vaccination date and the date of vaccine administration. In about one-third of 191 currently functional health districts, most deliveries occur in communities, and newborns are not brought to health facilities to be vaccinated and thus not considered when planning outreach vaccination sessions. In the same line, immigrants and emigrants are not taken into account when planning or monitoring health facility or outreach immunization sessions. Nomads move permanently from one village to another and are not targeted by immunization sessions. In some cases, nomads’ children receive several doses of the same vaccine at any time on their way, but none is recorded. This often leads to delaying or not vaccinating about 30%-70% of the targeted EPI population depending on the district. In 2004, we were able to improve the rates of timely immunizations of the third dose of the combined DPT-Hi+Hb vaccine in the Mada Health District in Far North-Cameroon. This was done in a year, using community volunteers to record the following information at the community level: births, travel of the EPI-targeted population, and immunization status of immigrant children. The resulting information was communicated to the vaccinating health facility to plan immunization sessions and organize the catch-up of those who missed the sessions. There was neither a control group nor sufficient power to draw a conclusion from this experience. To the best of our knowledge, this has not yet been tested. This project aims to test if using community volunteers to record vaccination status and demographic movement of children at the HH level and using the recorded data to plan immunization sessions and organize catch-up of missing children can improve EPI immunization timeliness, completeness, and coverage.

## Methods

### Trial Status

**Figure 1:**
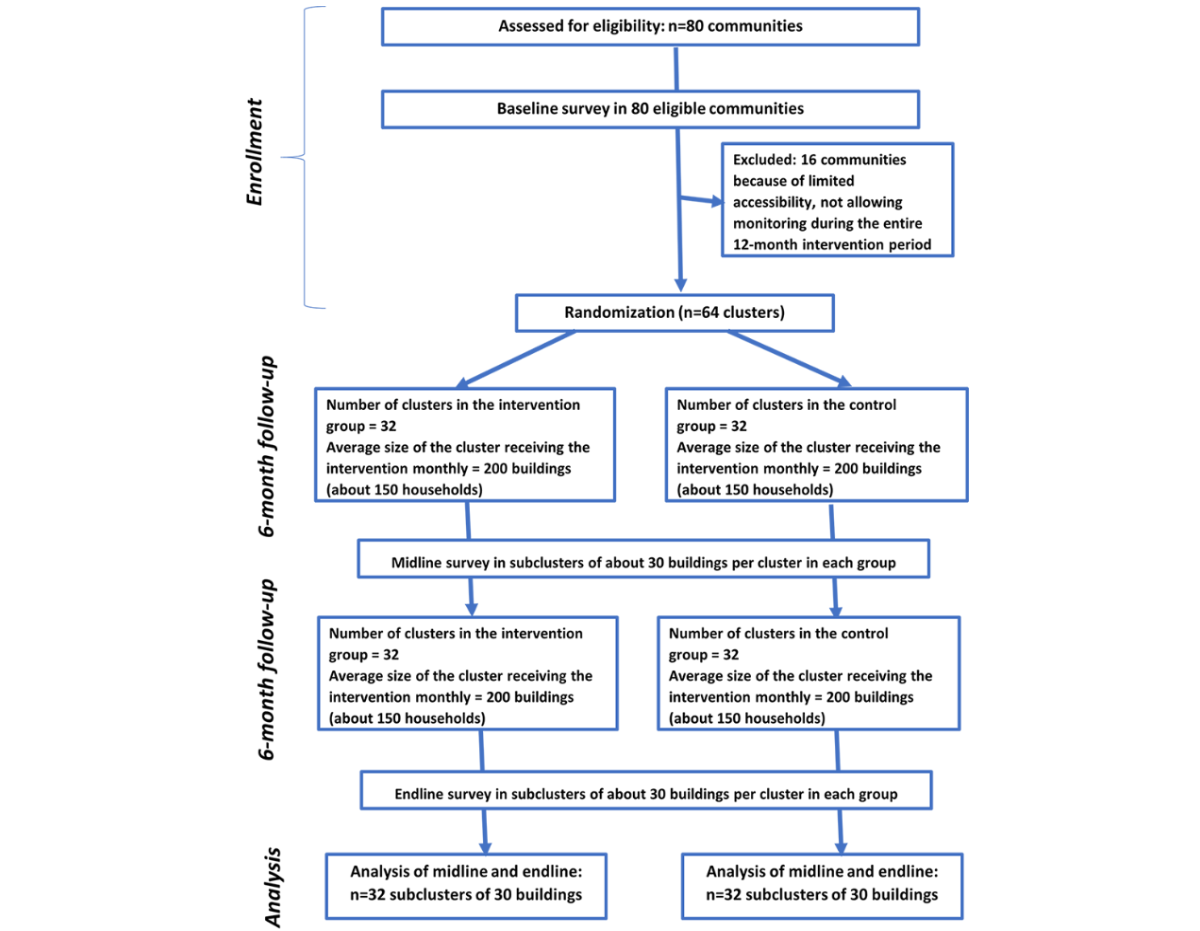
CONSORT diagram of the study.

The process of the field phase of the trial, which is ongoing, is described in Figure 1. The unique version of the protocol is registered in the Pan African Clinical Trials Registry with the number PACTR201808527428720. This protocol was also submitted to the Cameroon National Ethics committee for Human Health Research for ethical review, and after evaluation, ethical clearance (2018/07/1058/CE/CNERSH/SP) was obtained.

### Trial Design

This is a cluster randomized controlled trial in which communities of the targeted health district were randomly assigned to either the intervention or the control group. In the intervention group, community volunteers were trained to visit HHs monthly to record children’s immunization status and demographic movement in a community register, and the page of the register was scanned and sent to the health facility in charge of vaccination. The health facility vaccination team used the scanned page to plan where an outreach vaccination session was needed in the community. For the control group, EPI vaccination was organized as per routine, meaning on a weekly basis at health facilities for children living <5 km from the health facility or on a monthly basis during an outreach activity in the community for children situated ≥5 km away from the health facility.

### Study Site

**Figure 2:**
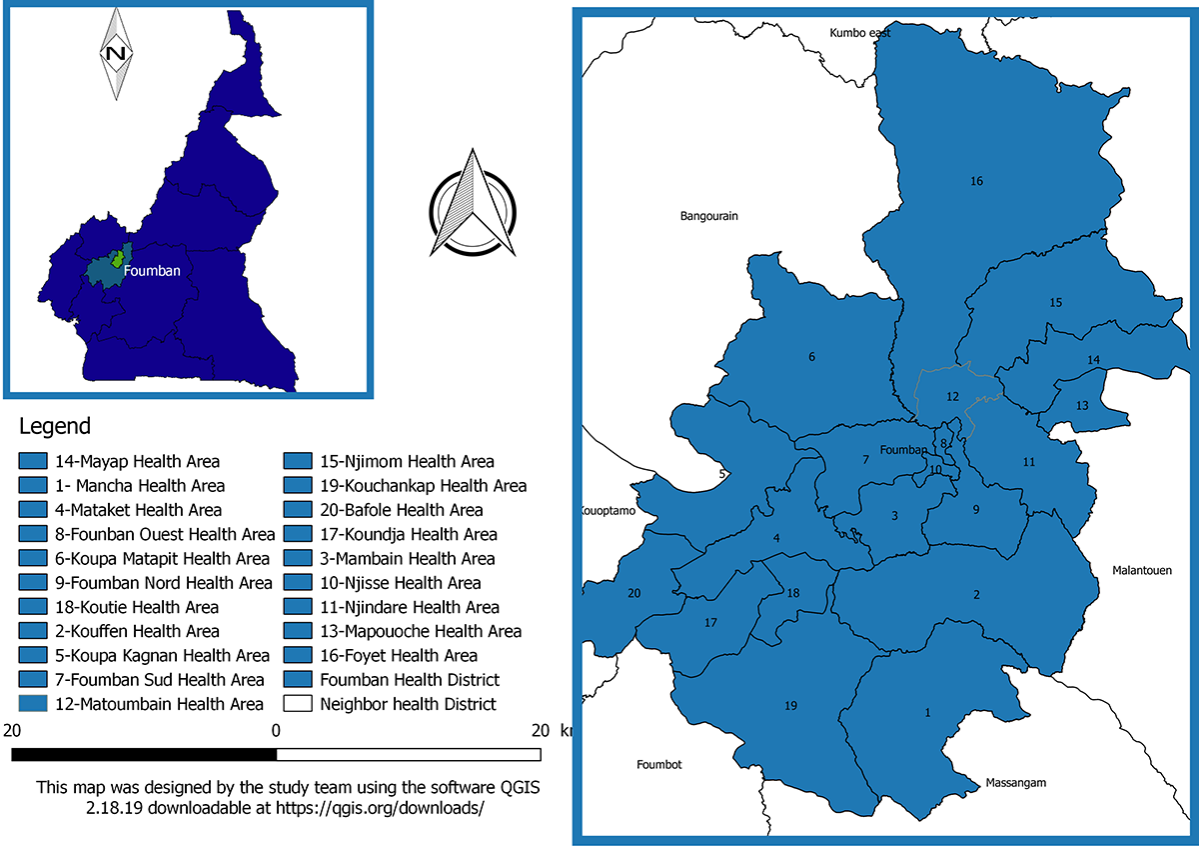
Map of the Foumban Health District.

The study was conducted in the Foumban Health District, West region of Cameroon, as shown in Figure 2. From the 2018 health population denominators of Cameroon, the total population of the district in 2018 was estimated at 235,828 inhabitants distributed in 311 communities [13]. This district is inhabited by a seminomadic population moving periodically each year with part or all their HH and cattle in search of pasture for farming activities. From weekly reports of the Epidemiological Surveillance Unit of the Department of Diseases Control, Cameroon Ministry of Public Health, it is one of the health districts that have been affected with at least one outbreak of EPI-PD during each of the previous 5 years.

### Study Period

This study was planned for a period of 18 months (from May 2018 to October 2019).

### Sampling

#### Sampling and Randomization of Clusters

To be eligible, communities had to have limited access to a vaccinating health facility and either have recorded a case of EPI-PD in the previous 12 months or belong to a health area with administrative DPT-Hi+Hb3 vaccine coverage <70% in the routinely EPI-targeted population. Communities with limited seasonal accessibility limiting the monitoring of the intervention in some period of the implementation of the intervention were excluded.

In this study, we considered a “community” to be the smallest geographic area (quarter) with a traditional leader (commonly called head of quarter) gathering 100-300 HHs in rural areas or 200-500 HHs in urban areas.

Selected communities were stratified according to their setting (urban/rural), the importance of yearly population movement, the distance to the vaccinating health facility, and the occurrence of EPI-PD in the previous year. In each stratum, communities were ranked in alphabetic order from A to Z and in blocks of 2. All combinations of blocks were listed, and a 1-digit number was assigned to each combination. Numbers were generated from Table XXXIII of Wishart [14], as follows: An arbitrary point was chosen in the table, and numbers were read in a single digit row by row across the page. Each number read and corresponding to a pair of communities dictated the distribution of these communities by study group. Randomized communities were divided into subunits of up to 200 buildings (ranging 100-150 HHs) called clusters using the Google Maps app installed on a smartphone. From previous experience, this is the number of buildings that can be visited in a week by a community volunteer to implement planned activities. One of these clusters per village was randomly chosen per village in each study group. To be able to compare immunization coverage and other indicators between baseline, midline, and endline surveys, eligible but not selected communities were similarly and independently randomized but did not receive the intervention.

#### Participants

All children aged 0-59 months living in HHs of the selected communities were eligible. This includes the age group targeted by the Cameroon national EPI for routine immunization (0-11 months) and the catch-up vaccination group program (12-59 months) [4]. Children arriving in a HH to stay for less than a month were excluded, and those leaving or planning to stay out of the HH for more than a month were not included. Those leaving the HH for less than a month were not excluded. Parents of children leaving the HH to stay for more than a month were followed up on phone when possible to sensitize them on the necessity of completing the child vaccination program.

### Intervention

One community volunteer per community was selected with the help of the head nurse of the competent health center (CHC) and trained to visit HHs of the community cluster monthly, record in a register all children aged 0-59 months and their demographic movements for the last month and next month, and assess their immunization status from the vaccination card or using a tracking grid if the child did not have a vaccination card. The recorded information page was snapped and sent through WhatsApp to the immunization team of the CHC. The information was used by the vaccination team that had received standardized training on reading and using WhatsApp images to plan and implement monthly community immunization sessions. This community vaccination session was conducted in collaboration with the community volunteers who chose an accessible vaccination site in the community and informed parents with children needing vaccination about the session. Health centers and communities were visited monthly to be supervised on their activities.

### Control

In the control group, immunization sessions were conducted as per routine. This meant the vaccination team organized weekly vaccination sessions at health facilities for villages situated <5 km away from the health center and when possible, monthly vaccination sessions in communities situated ≥5 km away.

### Outcomes Assessment

Data to assess effects of the interventions were collected from baseline, midline, and endline surveys. The baseline survey also provided data on population characteristics and child access to EPI vaccination prior to the intervention. Each village was mapped using the “my position” function of the Google Earth smartphone app and divided into clusters of about 30 buildings, assuming that each cluster would have at least 20 children aged 0-59 months (based on a pretest conducted in the area). One cluster was randomly selected per village and all its HHs visited for data collection. Data were collected by trained and supervised surveyors from the immunization card, community immunization register, and questionnaire administered to parents of children living in the village. Main data collected included the immunization status and timing regarding BCG, polio zero, DPT-Hi+Hb1, DPT-Hi+Hb2, and DPT-Hi+Hb3 and sociodemographic characteristics. The sampling and implementation process of the surveys were similar but independent [1]. The surveys were conducted by independent survey teams different from the team in charge of implementation of the intervention under investigation.

The primary outcome is the timeliness of documented immunization of children, defined as the proportion of children aged <5 years with documented BCG vaccination status administered within the first month of life.

Secondary outcomes include the documented completeness of general EPI vaccination of children, defined as the proportion of children who started vaccination with BCG and who completed it by receiving the measles-rubella vaccine, as documented on the immunization card, and documented completeness of specific immunization of children, defined as the proportion of children who received DPT-Hi+Hb1 and who completed pentavalent vaccination doses by receiving the DPT-Hi+Hb3 vaccine, as documented on the immunization card. In addition, we will assess the timeliness of overall immunization of children, defined as the proportion of children completing all their EPI-recommended vaccines within the first year of life, as documented on the immunization card or not documented but tracked from caregiver memory, and completeness of overall immunization of children, defined as the proportion of children who started vaccination with BCG and completed it by receiving the measles-rubella vaccine, as documented on the immunization card or not documented but tracked from caregiver memory. Documented coverage of child immunization is defined as the proportion of children who will have received DPT-Hi+Hb3, as documented on the immunization card, and overall coverage of child immunization is defined as the proportion of children who have received DPT-Hi+Hb3, as documented on the immunization card or tracked from caregiver memory. We will also assess the documented recruitment rate, defined as the proportion of children starting vaccination with BCG, as documented on the immunization card, and the overall recruitment rate, defined as the proportion of children starting vaccination with BCG, as documented on the immunization card or tracked from their mother’s memory.

The effects of the intervention will be assessed by comparing completeness, timeliness, and coverage estimated from the intervention and control groups.

### Sample Size Estimate

Using Stata software version 16.1 IC (StataCorp LLC, College Station, TX), the minimum required number of children to test the intervention was estimated at 20 children aged <5 years per cluster in at least 23 clusters of the control group and 20 in the intervention group. The estimate assumes between-cluster coefficients of variation of 0.38 and 0.19 in the control and intervention groups, respectively (estimated from baseline surveys in clusters assigned to each group), to reach 20 children who were <5 years old per cluster in each group, with assumptions that the proportion of children <5 years old vaccinated in the first month of life will remain at 20.5% (293/1430) based on a survey conducted in the targeted area prior to the intervention [1] and that an α error of .05 and 90% power will detect a 10% increase with the intervention in the proportion of children <5 years old vaccinated with BCG in the first month of their life. The estimate was guided by the method of estimating randomized controlled cluster trials proposed by Batistatou et al [15]. We adjusted to 32 clusters of at least 20 children per study group assuming 10% of the targeted children could not be reached (nonresponse and absence during the survey week) and in order to secure sufficient power to prevent cluster variation of estimated outcomes.

### Data Analysis

The effect of the intervention will be assessed by estimating per study group and comparing (1) yearly immunization timeliness rates for BCG vaccine (proportion of children aged 0-59 months with evidence of vaccination in the first month of life) and measles-rubella vaccine (proportion of children aged 12-23 months with evidence of vaccination while 9-11 months old); (2) coverage of BCG (proportion of children aged 0-59 months who were vaccinated) and DPT-Hi+Hb1, DPT-Hi+Hb3, and measles-rubella (proportion of children aged 12-59 months who were vaccinated) vaccines; and (3) specific completeness (proportion of children who received DPT-Hi+Hb1 and DPT-Hi+Hb3 vaccines) and general completeness (proportion of children who received BCG and measles-rubella vaccines) while aged 12-59 months. Odds ratios for children being vaccinated, being vaccinated on time, and completing vaccination will be estimated and adjusted for the child’s guardians’ level of education and profession, type of population (seminomadic or sedentary), distance to the vaccinating health facility, and religion. The odds ratios will be controlled for variability using logistic regression random effects. The fixed parameters will include the outcome, study groups, child’s guardians’ level of education, type of population (seminomadic or sedentary), child’s guardians’ profession, distance to the vaccinating health facility, and religion, and the random effect will be controlled on the participants’ cluster. The analysis will be done based on the intention to treat principle [16]. Data will be collected using Open Data Kit (ODK)–designed forms on smartphones, verified in the field, and submitted to a secure server. Data will be monitored and cleaned in Microsoft Excel 2013 and analyzed using Stata version 16.1 IC.

### Implementation Procedures

#### Procedures of Survey Implementation

GPS coordinates were collected at the limits of each selected cluster to map and retrieve the map on Google Earth. With the help of community volunteers, the cluster was divided into multiple subclusters of approximately 30 buildings each. One of the subclusters was randomly selected, and all the buildings and inhabited HH of these buildings were visited. All heads of HH were informed by the survey team about the project, and only consenting HHs were enrolled. In these HHs, data were collected from the caregiver for all children aged 0-59 months and from their immunization card about their immunization status as well as any demographic movement in the recent 6 months. The survey lasted for a week in each subcluster. Closed HH that were visited up to 3 times either on the same day or on 3 different days in this period were classified as closed. The study team arranged appointments with the head of HH and children’s guardian(s) if any were busy on the first day of the visit. Heads of HH and guardians who could not be met after 3 appointments on 3 different days were considered as nonrespondents. Children with caregivers refusing to respond to the questionnaire were not included, nor were children normally living in the HH but absent during the data collection period. 

The study questionnaires were pretested and developed into electronic forms by the data management team. Skip patterns and required and formatted fields were used to ensure data accuracy and completeness. Data were collected with smartphones using the ODK Collect application by trained surveyor teams. Each team of 3 surveyors was trained on the study procedures and supervised daily for participant sampling, informed consent, and data collection processes. A protected online server was deployed by the data management team to compile the survey data. During the survey, completed forms were uploaded to the server daily by the supervisor after reviewing and correcting discrepancies. The data management team ensured daily data cleaning, sharing of reports with field supervisors, and monitoring of corrections, updates, and backups. These procedures were the same in both study groups for the baseline, midline, and endline surveys.

#### Procedures for the Interventions

In the intervention group, one community volunteer living in selected communities was selected with the help of the head of the CHC and trained to collect data monthly per HH in the same periodicity over 12 months: demographic movements of children aged 0-59 months (births, deaths, immigration, emigration, travel, and short-term visits) and immunization status regarding each of the EPI vaccines. They assessed immunization status of all children aged 0-59 months staying in the visited HH or visiting for a stay of at least one week, or who stayed there in the previous month and had left definitely or for less than a week. Data were recorded in a designated register. The filled pages of the register were snapped and sent to the CHC through WhatsApp to the team in charge of vaccination. From a rapid analysis of received data, the nurse communicated by SMS to the community volunteer the list of children eligible for the monthly immunization session. The community volunteers informed the parents of these children about the place and time of the immunization session and why they should not miss the immunization session. The community volunteers educated all pregnant women on the importance of delivering in a health facility to increase the chance of the newborn being vaccinated. For births occurring in a community on a date far from the planned community immunization session, the community volunteer encouraged the mother to carry the newborn to the closest vaccinating health facility to be vaccinated. The community volunteer of this group had to contact any nomadic group crossing his community to list all children aged 0-59 months and collect data on their vaccination status. If there were children that needed to be vaccinated, the community volunteer completed the register and sent the page to the nurse in charge of vaccination in the community. A vaccination session was organized to vaccinate these children in case the nomad group had to leave the village before the monthly immunization session. The community volunteer was asked to collect telephone contacts from all parents who planned to travel from his community with the child. The child's parent was called by phone to be reminded to have his or her child vaccinated by the competent team.

During this period, EPI vaccination was delivered in the control group as per routine (ie, health facilities organized either the weekly vaccination session in health facilities for communities situated <5 km away from the health facility and monthly vaccination sessions in communities or monthly as an outreach activity in the communities). In each group, vaccination was recorded on a vaccination card and given to the children’s parents or guardians. Each community volunteer and one representative from each nomadic group had a register on which the immunization status of each child was recorded and updated after each immunization session and used to trace the immunization status in case the vaccination card was lost. A copy of the register was kept in the health facility, updated from the community register, and used to draft monthly immunization reports.

Health facilities in charge of vaccination in each of the communities organized routine vaccine supply to cover the vaccine needs in all study groups. Cold chain monitoring was also conducted as per routine. All health facilities were supervised regarding vaccine supply and cold chain monitoring. The community volunteers were supervised on a monthly basis to make sure they implemented the intervention as planned.

### Ethical Considerations

The present study is proposed to test an intervention that is expected to improve timely access to EPI vaccines by children in areas with frequent outbreaks of EPI-PD. It will involve interacting with communities, heads of HH, and caregivers to collect data on child vaccination status and demographic movements as well as organizing vaccination catch-ups. All local health, administrative, and traditional authorities with competency over the targeted study area were given information on the study, and they provided permission for study implementation. All caregivers were informed and provided consent for their participation and that of their children before being included in the study. For adults (≥21 years old), they were informed, and their consent required. For children (12-20 years old), their assent was required as well as parental consent for children aged <12 years. Surveillance of adverse events will be conducted routinely by the health facilities in charge. Data collected in registers for the monitoring of child vaccination will be shared between community volunteers and the health facility vaccination team but data extracted from these registers for the study purpose will be anonymous and stored in a secure database with access limited only to members of the study team. The protocol was evaluated and approved by the Cameroon National ethics committee (2018/07/1058/CE/CNERSH/SP), authorized by the Cameroon Ministry of Public Health (631-19-18), and registered in the Pan African Clinical Trials Registry (PACTR201808527428720; August 22, 2018). Relevant national and international regulations will be respected during the implementation of the protocol. Results of the study will be presented to representatives of targeted communities, community volunteers, local and ministerial health authorities, and scientists, hoping it will be used to improve children’s access to immunization.

## Results

This study is funded by the Bill and Melinda Gates Foundation under the 2018 Grand Challenges Opportunities round 20. As of January 2020, data collection was completed. Data cleaning and reporting are ongoing.

## Discussion

This project aims to assess the effect of community-based tracking and recording of child vaccination status and demographic movements and using these to plan immunization sessions and organize catch-up vaccination and to assess the effect on EPI immunization timeliness, completeness, and coverage. To the best of our knowledge, this is an innovative approach that has not yet been tested.

The EPI is proposed in almost all countries of the world to reduce the burden of EPI-PD in children [16]. Despite the fact that this program is supported by international and national organizations and institutions to be delivered free of charge to the targeted population, access to vaccines is still limited [17]. Many studies have described and documented the distribution, characteristics, and consequences of low EPI vaccine coverage and resulting EPI-PD [9]. Many interventions including organization of supplementary immunization campaigns, facility-based education, redesigned immunization reminder cards, health education in HHs and communities, regular immunization outreach, or integration of immunization with other services have improved children’s access to immunization but more is still required to achieve the objectives of EPI [11,12,18]. None of these studies assessed the effect of the interventions tested in this study on child vaccination timeliness, completeness, and coverage.

The intervention proposed in this study is expected to improve timely access by children to immunization since it includes collecting data from the community and making those data available to vaccination teams: reliable and documented data on children in need of vaccination on a monthly basis. Each HH was visited, and each child living in the HH, new birth, or impending child was recorded. In addition, each child’s immunization status was assessed. Each death or departing child was also recorded for better monitoring of vaccination. The study also provided an opportunity for children aged 12-59 months to complete missed EPI vaccine doses. Improving the timeliness and completeness of EPI vaccination will improve the coverage of children aged 0-11 months and 12-59 months with EPI-recommended doses of vaccines. The use of 2 vaccination registers, one of which is community-based and the other kept in the health facility, that are both simultaneously updated from HH visits and community vaccination sessions will help monitor the administration of subsequent immunization doses, thus improving documented vaccination timeliness, completeness, and coverage.

The tested intervention has some limitations, as its implementation will need additional resources. It requires that the involved health facilities have available health personnel in charge of vaccination and its supervision as well as transportation facilities for community immunization sessions. It also requires incentive and transportation costs for monthly HH visits by community volunteers. In low- and middle-income countries, which are expected to benefit the most from the intervention, these resources are not always sufficient to cover resources needed for the intervention. Since these resources can be acquired through funding, the study will assess the costs of an administered vaccination dose and compare it to the costs of other proposed interventions as immunization campaigns implemented to catch-up with missed vaccination doses. This will be used to inform decision makers and funders of immunization activities to guide the decision-making process in choosing better, cost-effective vaccination interventions.

## Abbreviations

BCG: bacille Calmette-Guérin
CHC: competent health center
DPT-Hi+Hb: Diphtheria-Pertussis-Tetanus and Hepatitis B + Hemophilus Influenzae type b
EPI: Expanded Programme on Immunization
EPI-PD: EPI vaccine–preventable diseases
HH: household
ODK: Open Data Kit
